# Emphysematous Gastritis: A Real Indication for Emergent Surgical Intervention?

**DOI:** 10.7759/cureus.8106

**Published:** 2020-05-13

**Authors:** Sana Riaz, Pujitha Kudaravalli, Sheikh A Saleem, Bishnu Sapkota

**Affiliations:** 1 Internal Medicine, State University of New York Upstate Medical University, Syracuse, USA; 2 Gastroenterology, State University of New York Upstate Medical University, Syracuse, USA

**Keywords:** emphysematous gastritis, epigastric pain, coffee-ground emesis, conservative management

## Abstract

Emphysematous gastritis is a rare disease with gastric inflammation and intramural gas formation due to gas-forming microorganisms. It is diagnosed based on clinical presentation and imaging findings of gas in the gastric wall. Computed tomography is the preferred imaging modality. Early diagnosis and management are important since emphysematous gastritis is associated with high rates of morbidity and mortality. We present a case of emphysematous gastritis, which was successfully managed conservatively through early diagnosis and prompt treatment.

## Introduction

Emphysematous gastritis is a rare, life-threatening form of gastritis caused by gas-forming microorganisms, which has a high mortality rate of 55%-61% [1,2]. Emphysematous gastritis is associated with alcohol abuse, diabetes mellitus, renal failure, recent abdominal surgery, gastroenteritis, long-term corticosteroid use, ingestion of corrosive agents, and nonsteroidal anti-inflammatory drug use [1]. Microorganisms associated with emphysematous gastritis include *Streptococcus *species*, Escherichia coli, Enterobacter *species*, Clostridium *species*, Pseudomonas aeruginosa, Staphylococcus aureus, Candida *species*, *and *Mucor* species [3]. Only 59 cases have been reported in the English language literature based on a review by Watson et al., and no guidelines are available for the management of emphysematous gastritis [2]. However, early diagnosis and initiation of medical management with bowel rest, hydration, and intravenous broad-spectrum antibiotics have been noted to improve outcomes. Surgical intervention is not indicated during acute infection and is reserved for patients who have failed optimal medical management, demonstrating signs of clinical deterioration, perforations, peritonitis, and uncontrolled disseminated sepsis [3]. Limiting surgical intervention during acute infection is important to decrease the occurrence of anastomotic leak, postoperative fistulas, and strictures [2].

## Case presentation

**Figure 1:**
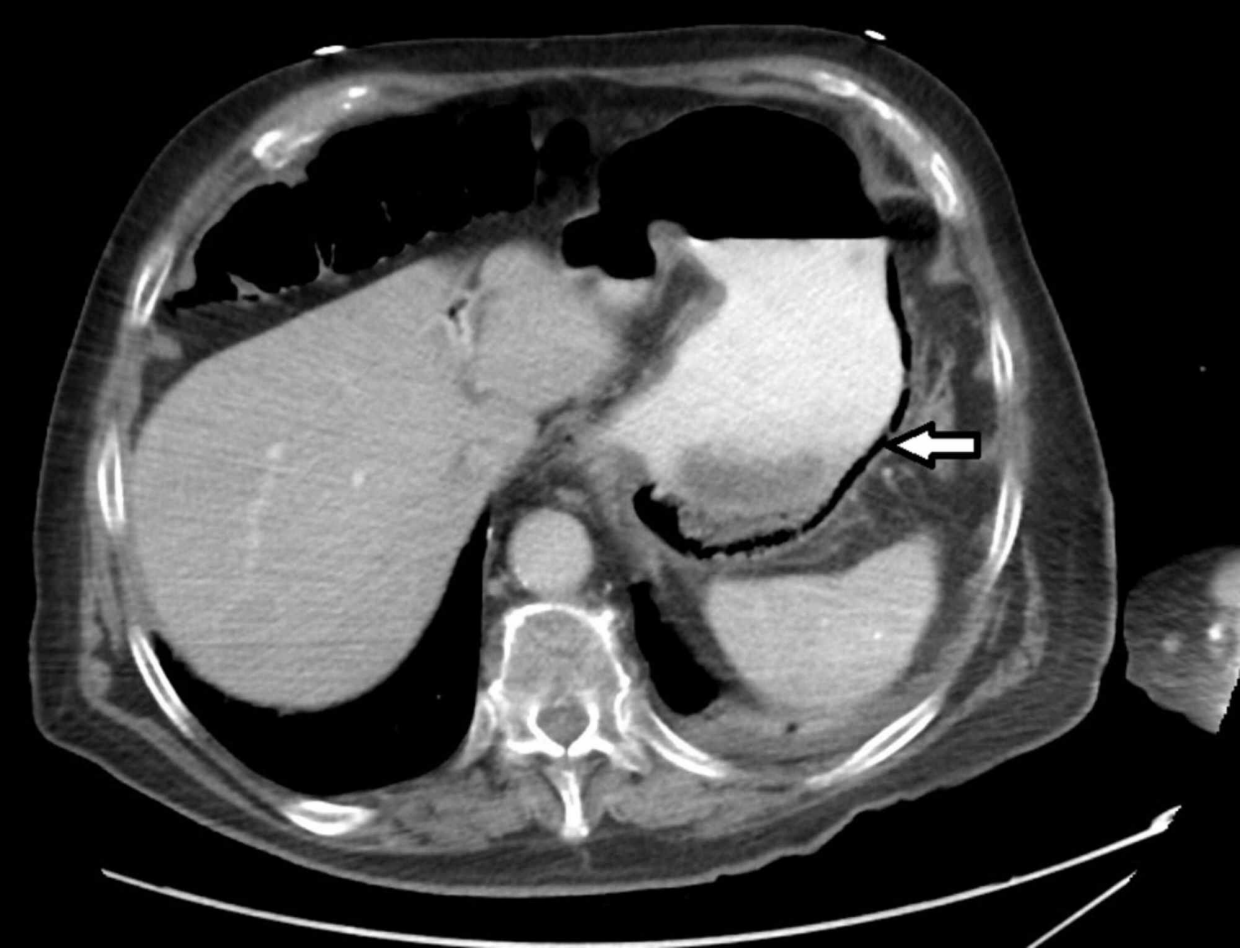
Computed tomography of the abdomen and pelvis showing gastric wall thickening along with circumferential gas formation (arrow).

A 96-year-old male with a history of bioprosthetic aortic valve on aspirin and warfarin, hypertension, dyslipidemia, and prostate cancer presented with acute-onset epigastric discomfort, coffee-ground emesis, and melena. He was a non-smoker and non-alcoholic. On presentation, he was hemodynamically stable with blood pressure 102/77 mmHg, pulse 75 beats per minute, respiratory rate 18 breaths per minute, and temperature 36.5°C. Physical examination was notable for epigastric tenderness and black tarry stool and per rectal exam. Laboratory studies showed leukocytosis (white blood cell count of 11.8 x 10^3^/µL), thrombocytopenia of 108 x 10^3^/µL (reference range 150-400 x 10^3^/µL), and elevated blood urea nitrogen of 44 mg/dL (reference range 6-20 mg/dL) with normal creatinine level. Blood cultures were unremarkable. He underwent computed tomography (CT) of the abdomen; pertinent imaging findings included gastric wall thickening along with gas (Figure 1). 

**Figure 2:**
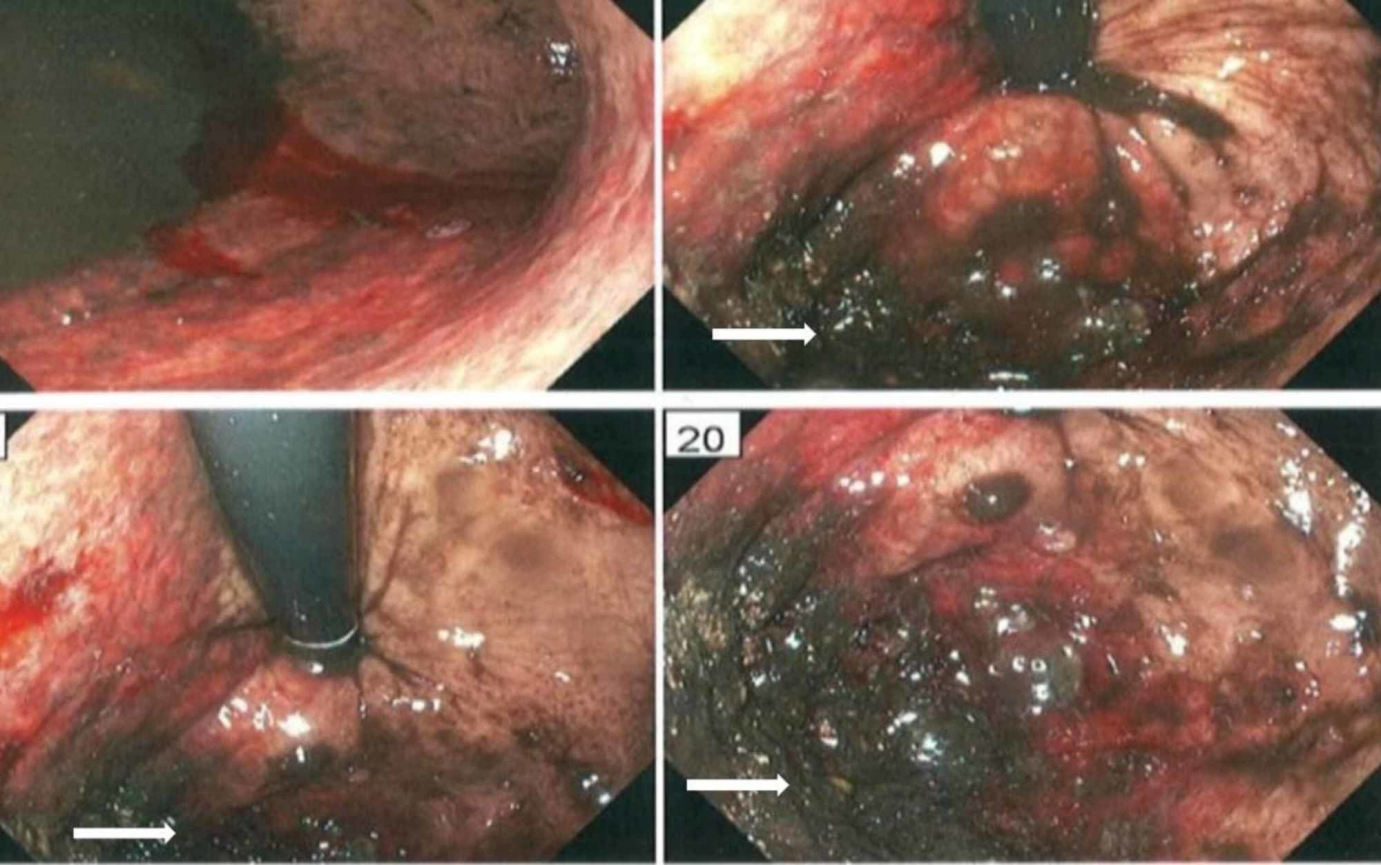
Esophagogastroduodenoscopy showing gastric wall necrosis along the greater curvature (arrows).

**Figure 3:**
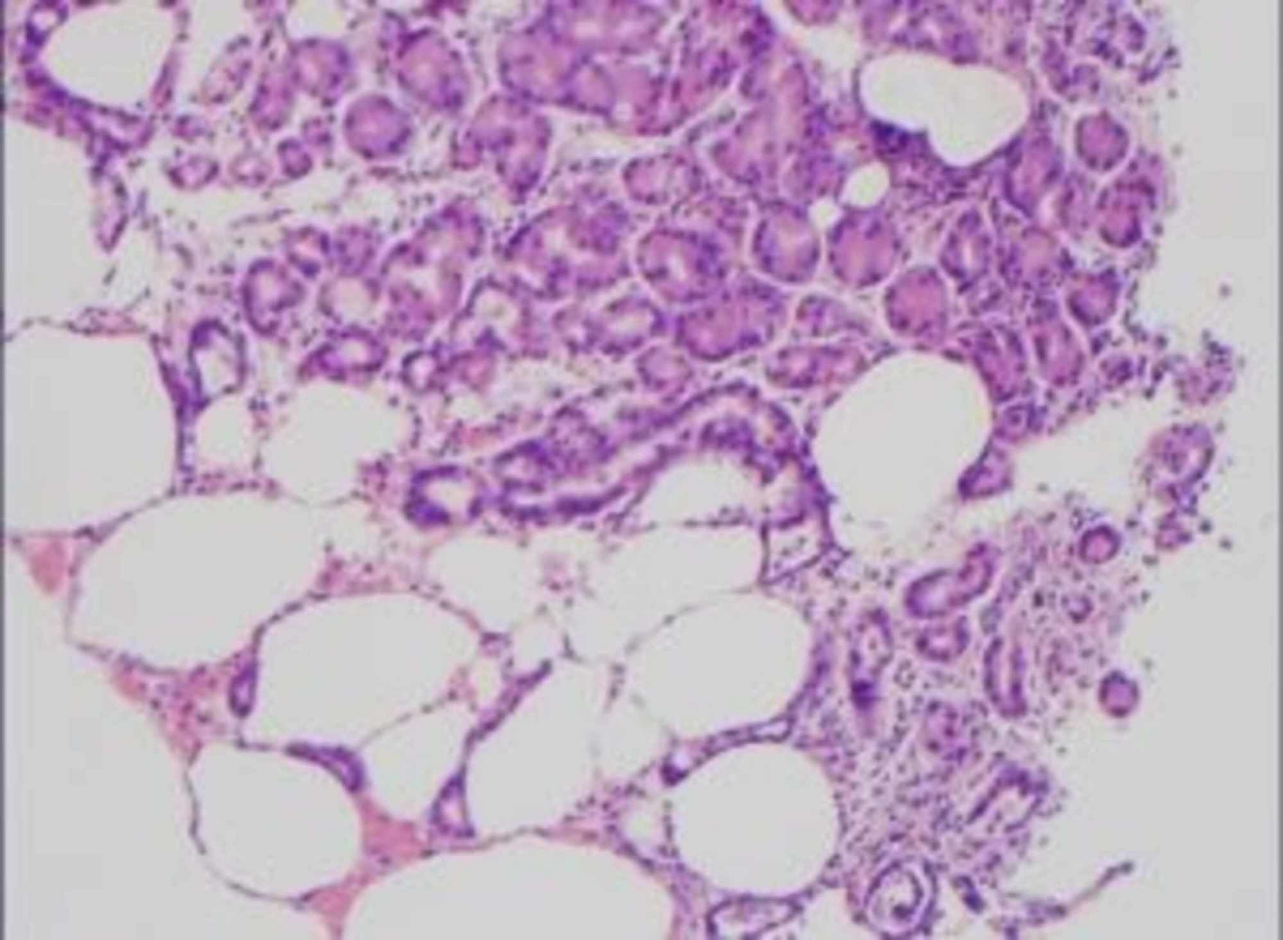
Pathology slide demonstrating emphysematous gastritis.

Additional evaluation with esophagogastroduodenoscopy and biopsy showed findings consistent with gastric wall necrosis (Figures 2, 3). He was medically managed with nasogastric tube gastric decompression, intravenous pantoprazole, broad-spectrum intravenous antibiotics (vancomycin, and piperacillin-tazobactam), and intravenous fluids. The patient did very well with medical management. An improvement was noted within 48 hours, and he was subsequently discharged home in stable condition, 10 days after hospitalization. 

 

## Discussion

The differential diagnoses for intramural gas in the stomach include gastric emphysema and emphysematous gastritis. It is essential to differentiate the two entities, which are characterized by different clinical symptoms, radiological findings, treatment, and prognosis. Gastric emphysema is a benign process and is usually associated with gastric outlet obstruction, excessive vomiting, nasogastric tube placement, and cardiopulmonary resuscitation. Acute abdominal symptoms are absent in gastric emphysema, and imaging is notable for the linear distribution of gas in the gastric wall. It resolves spontaneously without treatment [4]. 

In contrast to gastric emphysema, emphysematous gastritis is caused by a bacterial infection that may arise locally through the mucosa or hematogenous spread from a distant focus [5]. Patients usually present with severe abdominal pain, nausea, vomiting (occasionally hematemesis), and fever. Physical examination findings include abdominal distension and decreased bowel sounds. CT is the preferred and most effective diagnostic imaging modality. Pertinent imaging findings for emphysematous gastritis include gastric wall thickening and presence of irregular, mottled gas in the stomach wall, particularly in the fundus and greater curvature [1,6]. These findings remain in place despite changes in body position [6]. 

There are no diagnostic criteria for detecting emphysematous gastritis; however, based on previous studies, it is diagnosed on the following criteria: (1) clinical presentation, (2) inflammatory response based on blood tests, (3) imaging findings, and (4) evidence of bacterial infection, based on gastric fluid culture or pathology specimens [1]. The factors associated with higher mortality include elevated serum lactate and creatinine levels and concomitant pneumatosis in small bowel and colon, thereby requiring higher vigilance and management [7].

Medical management is the therapeutic option in patients with emphysematous gastritis. Emergent surgery is indicated in patients with deterioration despite optimal medical management, the involvement of a large portion of the stomach, presence of gastric infarction, or perforation [6]. Based on a study by Watson et al., it was noted that fewer patients with emphysematous gastritis underwent surgical intervention after the year 2000 (62.5% before 2000 versus 22.2% after 2000, P = 0.002) and were associated with a lower mortality rate (59.4% before 2000 versus 33.3% after 2000, P = 0.046) [8]. 

Through our case, we want to highlight that emphysematous gastritis can be successfully managed conservatively without the need for surgery, especially in elderly patients with multiple comorbidities similar to our patient.

The case has previously been submitted as an abstract to the American Council of Gastroenterology Conference [9].

## Conclusions

Emphysematous gastritis is a severe form of gastritis and has a poor prognosis. Early diagnosis and management are imperative to improve outcomes. Our patient, who is an elderly gentleman with multiple comorbidities, was successfully managed with medical therapy, thereby suggesting that surgical intervention for emphysematous gastritis should not be used as initial therapy, and should be considered when medical management has failed. 

## References

[1] Takano Y, Yamamura E, Gomi K (2015). Successful conservative treatment of emphysematous gastritis. Intern Med.

[2] Watson A, Bul V, Staudacher J, Carroll R, Yazici C (2017). The predictors of mortality and secular changes in management strategies in emphysematous gastritis. Clin Res Hepatol Gastroenterol.

[3] Sharma P, Akl EG (2016). A combination of intramural stomach and portal venous air: conservative treatment. J Community Hosp Intern Med Perspect.

[4] Yalamanchili M, Cady W (2003). Emphysematous gastritis in a hemodialysis patient. South Med J.

[5] Loi T-H, See J-Y, Diddapur RK, Issac JR (2007). Emphysematous gastritis: a case report and a review of literature. Ann Acad Med Singapore.

[6] Jung JH, Choi HJ, Yoo J, Kang SJ, Lee KY (2007). Emphysematous gastritis associated with invasive gastric mucormycosis: a case report. J Korean Med Sci.

[7] Jehangir A, Rettew A, Shaikh B, Bennett K, Qureshi A, Jehangir Q (2015). A case report of emphysematous gastritis in a diabetic patient: favorable outcome with conservative measures. J Community Hosp Intern Med Perspect..

[8] Nasser H, Ivanics T, Leonard-Murali S, Shakaroun D, Woodward A (2019). Emphysematous gastritis: a case series of three patients managed conservatively. Int J Surg Case Rep.

[9] Riaz S, Kudaravalli P, Saleem SA, Sapkota B (2019). Emphysematous gastritis: a real indication for emergent surgical intervention?. Am J Gastroenterol.

